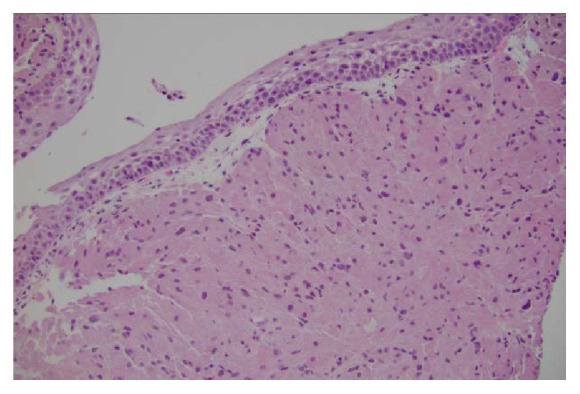# Multifocal Pulmonary Granular Cell Tumor Presenting with Postobstructive Pneumonia

**DOI:** 10.1155/2017/8513702

**Published:** 2017-10-15

**Authors:** Samid M. Farooqui, Muhammad S. Khan, Laura Adhikari, Viral Doshi

**Affiliations:** ^1^Department of Internal Medicine, University of Oklahoma Health Sciences Center, Oklahoma City, OK, USA; ^2^Section of Pulmonary, Critical Care and Sleep Medicine, University of Oklahoma Health Sciences Center, Oklahoma City, OK, USA; ^3^Department of Pathology, University of Oklahoma Health Sciences Center, Oklahoma City, OK, USA

## Abstract

Granular cell tumor (GCT) is a neoplasm of Schwann cell origin. Its presence in the aerodigestive tract is uncommon and becomes a diagnostic challenge on initial presentation. Our case is of a 59-year-old woman who presented to the emergency department with a history of productive cough and dyspnea associated with fever and chest pain. An initial chest X-ray (CXR) showed a right middle lobe consolidation with follow-up Computed Tomography (CT) scan showing a mass in the right bronchus. Bronchoscopy revealed a polypoid, sessile granular mass in the right bronchus intermedius with multiple white lesions in trachea, left main bronchus, and right upper bronchi. Histology revealed a benign GCT. Bronchoalveolar lavage from the right middle lobe grew* Streptococcus pneumoniae*. Patient was treated with intravenous levofloxacin during hospital stay and discharged on a 7-day course of oral antibiotics to be followed as outpatient but was lost to follow-up. GCT can present as a polypoid tumor causing recurrent postobstructive pneumonia. Surgical resection is the most successful treatment option. The tumor is more common in third and fourth decade of life and our patient is the oldest patient, according to our knowledge, to have a GCT.

## 1. Introduction

Granular cell tumor (GCT) is a rare lesion of Schwann cell origin which frequently occurs in the upper aerodigestive tract but can potentially affect all parts of the body, including the skin. The head and neck area is affected 45%–65% of the time with more than two-thirds of the lesions occurring intraorally—tongue, mucosa and hard palate being the most common sites [1]. Involvement of the bronchi and lower respiratory tract is much less common, with the lesions typically being solitary, submucosal origin nodules [2]. We present here the case of a rare large polypoid GCT causing postobstructive pneumonia.

## 2. Case

**Figure 1:**
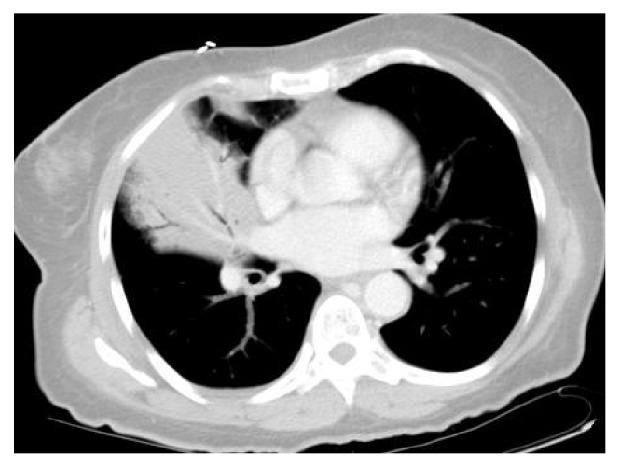


**Figure 2:**
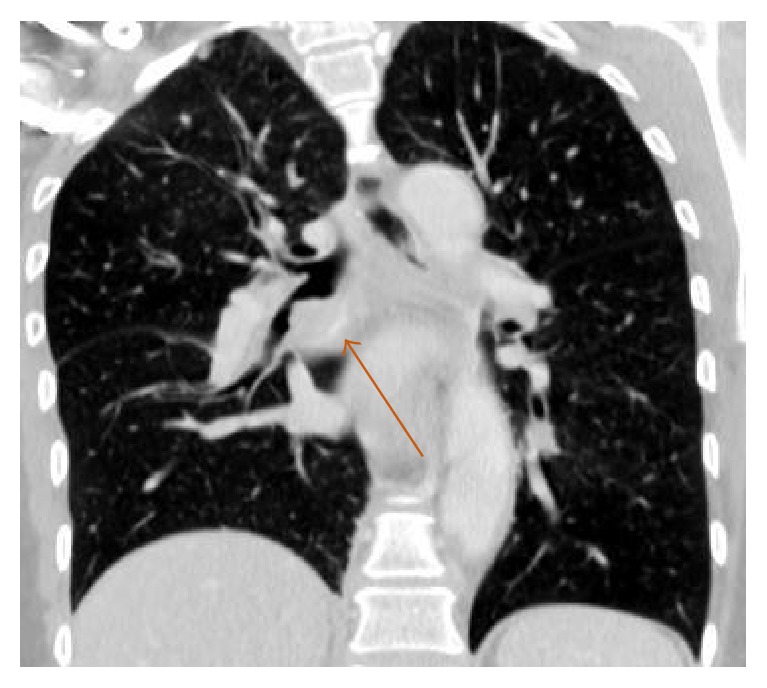


Our patient is a 59-year-old African American female with a past medical history of asthma who presented to the emergency department with shortness of breath, fever, and persistent right-sided chest pain with a cough productive of purulent sputum for the last 1 week. Physical examination was unremarkable except for crackles in the right lung base. Blood workup was significant for leukocytosis of 28,760/mm^3^ (4.00–11.00 K/mm^3^), with normal hemoglobin level, platelet count, and chemistry panel. Chest X-ray (CXR) performed was significant for a right middle lobe consolidation. A subsequent CT chest showed a right middle lobe consolidation, with an endobronchial lesion within the right bronchus intermedius, concerning for postobstructive pneumonia (Figures 1 and 2). She was started on levofloxacin for treatment and admitted for further workup.

**Figure 3:**
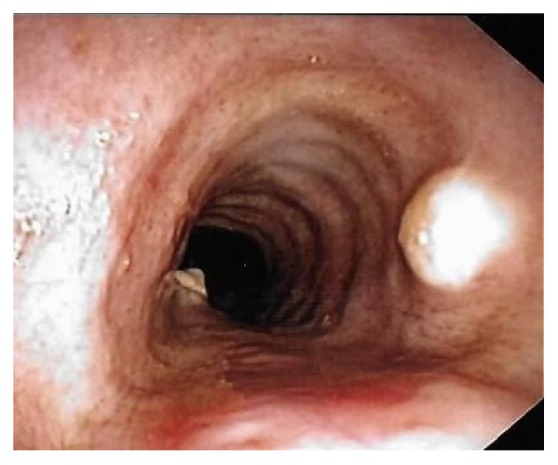


**Figure 4:**
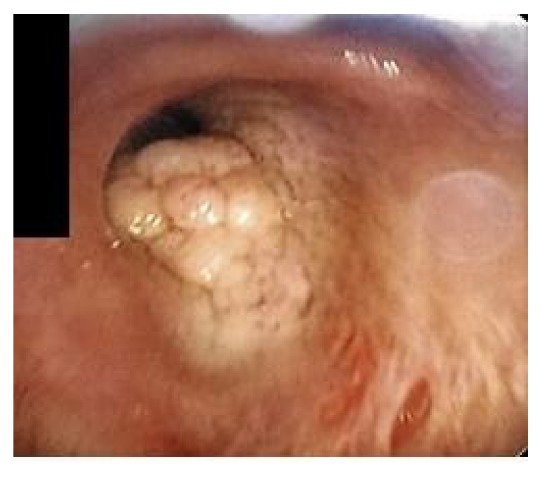


**Figure 5:**
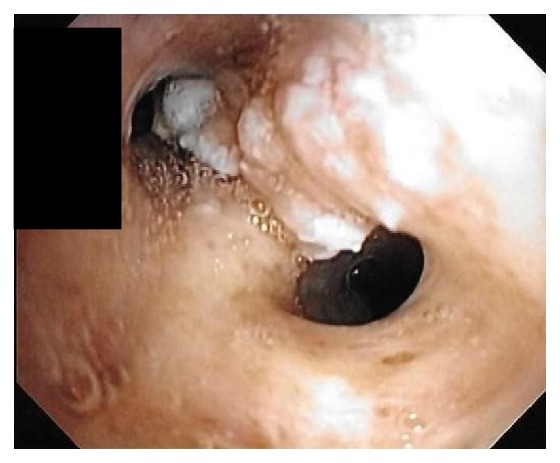


**Figure 6:**
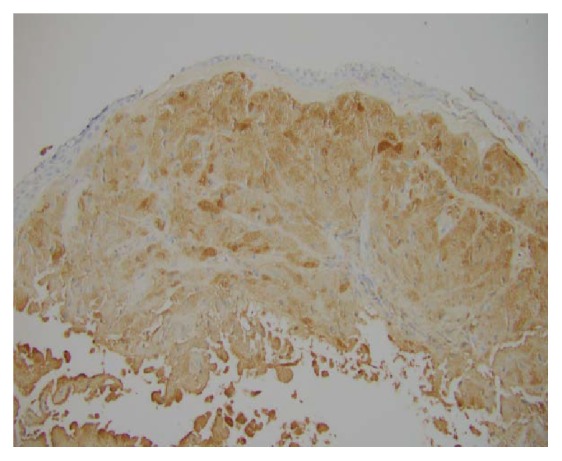


**Figure 7:**
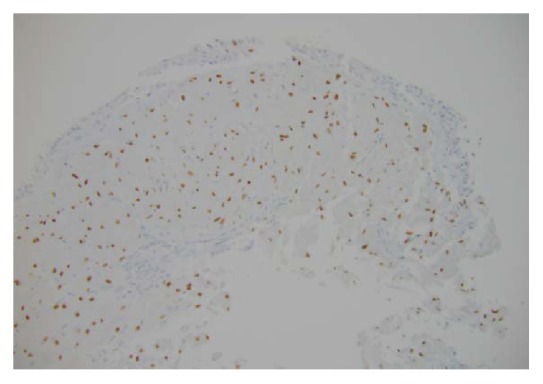


A bronchoscopy was performed which showed multiple flat white colored lesions in the trachea, left main-stem bronchi, right upper lobe bronchi (Figure 3), and a large polypoid, sessile, and white colored tumor located just distal to the secondary carina causing near-complete obstruction (>80%) of the right proximal bronchus intermedius (Figures 4 and 5). Several endobronchial biopsies were obtained from the large polypoid tumor located in bronchus intermedius and from the lesion in left main bronchus. Histopathological examination was consistent with granular cell tumor with tumor markers S-100 (Figure 6), Sox-10 (Figure 7), and CD-68 positive. Immunohistochemical analysis for pancytokeratin, cytokeratin 8/18, and synaptophysin was negative. Notably, sputum culture and right middle lobe bronchoalveolar lavage culture grew* Streptococcus pneumoniae. *The patient improved over the course of 4 days and was discharged home with a course of oral antibiotics for a total of 7 days, and follow-up was arranged in pulmonary clinic. However, patient was lost to follow-up.

## 3. Discussion

Granular cell tumors (GCTs), first described by Abrikosov in 1926 [3], are now believed to be of neurogenic (Schwann cell) origin [4]. Pulmonary GCTs comprise approximately 6–10% of all GCTs with majority being found in the lower respiratory tract as polypoid, endobronchial masses [2]. The tumor commonly occurs in middle-aged patients, with mean age reported to be in the fourth decade of life (36-37 years) and an equal prevalence in males and females [5]. Our patient is the oldest individual reported in literature, per our knowledge. Common symptoms at presentation include hemoptysis (17%), chronic cough (13%), chest pain (6%), and unexplained dyspnea (3%). Patients can also present with an asymptomatic lung nodule (8%) on imaging or as an incidental finding on bronchoscopy (8%) [6]. Our patient presented with multiple respiratory tract GCTs, including a large polypoid granular cell tumor causing postobstructive pneumonia with cultures positive for* Streptococcus pneumoniae*.

Pulmonary GCTs, like their extrapulmonary counterparts, can rarely be multifocal and have been reported to be associated with genetic mutations in PTPN11 as part of LEOPARD syndrome [2, 7]. In one case series, multifocal GCTs were reported to arise from all lobes of the lung and from the main-stem bronchi [2]. In up to 25% of cases there can be multiple GCTs, but the presence of multifocal GCT in lung does not necessarily indicate malignancy [2]. The presence of metastatic malignant GCT from extrapulmonary sites has been described in the settings of multifocal pulmonary GCT [8, 9] and metastasis should be ruled out. In our patient, despite the presence of multifocal GCT in the lungs, CT scan of chest abdomen and pelvis did not reveal any extrapulmonary focus of the disease decreasing suspicion for metastasis.

The infiltrative nature of pulmonary GCTs is a well-established feature for this benign tumor [2]. Peribronchial tissue extension has been reported in up to 40% of tumors as these tumors like to grow along muscle fibers, fibrous septa, and nerve sheath bundles [2]. Pseudoepitheliomatous hyperplasia, an overgrowth or thickening of the overlying squamous epithelium (Figure 8), is a diagnostic feature seen for these tumors. Microscopically, the tumor is composed of abundant eosinophilic granular cytoplasm, with fairly uniform arrangement of small nuclei. GCT is mostly benign, with malignant course occurring in 2% of cases [10]. Differentiation between benign and malignant GCTs is often difficult. Six histologic features have been described which can predict malignant potential of GCTs. These features include spindling of the tumor cells, the presence of vesicular nuclei with large nucleoli, increased mitotic rate (>2 mitoses/10 high-power fields at 200x magnification), a high nuclear-to-cytoplasm (N : C) ratio, pleomorphism, and necrosis. Histologically, malignant GCT is diagnosed when three or more of the six criteria are fulfilled [11]. Our case did not fulfill the criteria for malignant GCT.

Treatment of patients with endobronchial GCT has not been clearly defined. Current therapeutic options include surgical resection, endoscopic removal, YAG laser, and fulguration [2]. Overall, surgical excision has the highest cure rate. Of the 20 surgically treated patients followed for a mean of 3.3 years, only one patient was reported to have had symptomatic recurrence. The extent of surgical resection is unclear; however most authors agree that when postobstructive parenchymal damage has occurred, segmental or lobar resection is indicated [6]. Sleeve resection is considered when local resection of a mass is anatomically feasible [2]. The tumors can be observed in some cases. Spontaneous resolution has been documented in only one case. If distal lung parenchyma is preserved, then bronchoscopic extirpation and laser therapy can be considered [2].

GCT can be associated with other neoplasms in approximately 13% of cases [12]. The most common neoplasm associated with pulmonary GCT is lung carcinoma. Esophageal cancer and renal cell carcinomas have also been observed in patients with pulmonary GCT [2]. Nonneoplastic diseases reported in patients with pulmonary GCT include sarcoidosis and HIV infection [1, 13].

In conclusion, pulmonary GCT is a rare entity, which can present as a large polypoid tumor causing recurrent postobstructive pneumonia and can be found throughout the bronchial tree and in peripheral lung fields in the form of multifocal GCTs, as in our case. Even though risk of malignancy is very rare, if multiple lung lesions are present, metastatic GCT should be ruled out by appropriate imaging. The patients should be followed at regular interval to assess for recurrence.

## Figures and Tables

**Figure 8:**